# Assessing Neonatal Intensive Care Unit Structures and Outcomes Before and During the COVID-19 Pandemic: Network Analysis Study

**DOI:** 10.2196/27261

**Published:** 2021-10-20

**Authors:** Hannah Mannering, Chao Yan, Yang Gong, Mhd Wael Alrifai, Daniel France, You Chen

**Affiliations:** 1 Department of Computer Science Loyola University Baltimore, MD United States; 2 Department of Computer Science Vanderbilt University Nashville, TN United States; 3 School of Biomedical Informatics The University of Texas Health Science Center at Houston Houston, TX United States; 4 Department of Pediatrics Vanderbilt University Medical Center Nashville, TN United States; 5 Department of Anesthesiology Vanderbilt University Medical Center Nashville, TN United States; 6 Department of Biomedical Informatics Vanderbilt University Medical Center Nashville, TN United States

**Keywords:** neonatal intensive care unit, collaboration, health care organization structures, intensive care, length of stay, discharge dispositions, electronic health records, network analysis, COVID-19, temporal network analysis

## Abstract

**Background:**

Health care organizations (HCOs) adopt strategies (eg. physical distancing) to protect clinicians and patients in intensive care units (ICUs) during the COVID-19 pandemic. Many care activities physically performed before the COVID-19 pandemic have transitioned to virtual systems during the pandemic. These transitions can interfere with collaboration structures in the ICU, which may impact clinical outcomes. Understanding the differences can help HCOs identify challenges when transitioning physical collaboration to the virtual setting in the post–COVID-19 era.

**Objective:**

This study aims to leverage network analysis to determine the changes in neonatal ICU (NICU) collaboration structures from the pre– to the intra–COVID-19 era.

**Methods:**

In this retrospective study, we applied network analysis to the utilization of electronic health records (EHRs) of 712 critically ill neonates (pre–COVID-19, n=386; intra–COVID-19, n=326, excluding those with COVID-19) admitted to the NICU of Vanderbilt University Medical Center between September 1, 2019, and June 30, 2020, to assess collaboration between clinicians. We characterized pre–COVID-19 as the period of September-December 2019 and intra–COVID-19 as the period of March-June 2020. These 2 groups were compared using patients’ clinical characteristics, including age, sex, race, length of stay (LOS), and discharge dispositions. We leveraged the clinicians’ actions committed to the patients’ EHRs to measure clinician-clinician connections. We characterized a collaboration relationship (tie) between 2 clinicians as actioning EHRs of the same patient within the same day. On defining collaboration relationship, we built pre– and intra–COVID-19 networks. We used 3 sociometric measurements, including eigenvector centrality, eccentricity, and betweenness, to quantify a clinician’s leadership, collaboration difficulty, and broad skill sets in a network, respectively. We assessed the extent to which the eigenvector centrality, eccentricity, and betweenness of clinicians in the 2 networks are statistically different, using Mann-Whitney *U* tests (95% CI).

**Results:**

Collaboration difficulty increased from the pre– to intra–COVID-19 periods (median eccentricity: 3 vs 4; *P*<.001). Nurses had reduced leadership (median eigenvector centrality: 0.183 vs 0.087; *P*<.001), and neonatologists with broader skill sets cared for more patients in the NICU structure during the pandemic (median betweenness centrality: 0.0001 vs 0.005; *P*<.001). The pre– and intra–COVID-19 patient groups shared similar distributions in sex (~0 difference), race (4% difference in White, and 3% difference in African American), LOS (interquartile range difference in 1.5 days), and discharge dispositions (~0 difference in home, 2% difference in expired, and 2% difference in others). There were no significant differences in the patient demographics and outcomes between the 2 groups.

**Conclusions:**

Management of NICU-admitted patients typically requires multidisciplinary care teams. Understanding collaboration structures can provide fine-grained evidence to potentially refine or optimize existing teamwork in the NICU.

## Introduction

Health care organizations (HCOs) change intensive care unit (ICU) staffing and follow physical distancing policy during the COVID-19 pandemic to protect clinicians and patients [1,2]. For instance, many physical care activities before the COVID-19 pandemic have been transitioning to virtual systems, such as electronic health records (EHRs) or telehealth [3-5]. These changes can interfere with the structures of teamwork in the ICU, which may impact clinical outcomes. The changes in ICU structures and outcomes from pre– to intra–COVID-19 periods have not been systematically investigated. Therefore, challenges are unclear when health care delivery disruptions (eg, pandemics) or major transitions (physical to virtual collaboration) occur in the post–COVID-19 era.

One of the major challenges to analyzing ICU structures and quantifying their changes is that the ICU structures are historically developed at a coarse-grained level, which seldom considers connections among clinicians in a team owing to dynamic and complex clinical workflows, shifts, and handovers [6-9]. Understanding how clinicians connect (eg, sharing and exchanging health information) within their clinical teams when caring for patients can provide fine-grained evidence to potentially refine or optimize existing ICU structures.

In modern health care, an increasing number of clinicians utilize EHR to diagnose and treat patients by exchanging all medical statuses [10,11]. Therefore, the volume of the EHR system utilization data has been increasing exponentially in recent years, providing abundant resources to identify connections between clinicians. Recent studies applied network analysis to EHR utilization data to measure connections among clinicians [12-15]. They found EHR system utilization data to potentially be a rich resource to be leveraged to model relationships among clinicians. Recent studies have also shown that network analysis methods and data within the EHR can also be utilized to learn collaboration structures in ICUs [8,9,16]. Based on previous studies, this study leverages network analysis methods to learn structures of the neonatal ICU (NICU) in pre– and intra–COVID-19 eras in terms of collaboration among clinicians, and compares differences in the structures. Patients hospitalized in the NICU include high-risk infants who may be or are at risk for a variety of complex diseases or conditions. The management of NICU patients typically requires multidisciplinary care teams (eg, neonatal frontline providers, ancillary staff, nurses, neonatologists, residents, support staff, respiratory therapists, neonatal fellows, and highly specialized consultants) [17-19]. We investigate the connections among clinicians in a tertiary-level NICU, which has a high density of intense EHR utilization and heavy data sharing traffic per patient episode [8,9], making this environment ideal for our ICU structure study.

## Methods

To describe our work systematically, we used the reporting checklist for quality improvement in health care (Multimedia Appendix 1), which is based on the SQUIRE 2.0 guidelines [20].

We extracted EHRs for all patients admitted to the NICU at Vanderbilt University Medical Center (VUMC, Nashville, Tennessee) between September 1, 2019, and June 30, 2020. We characterized pre–COVID-19 as the period of September through December 2019 and intra–COVID-19 as the period of March through June 2020. We used network analysis methods to analyze the EHRs of 712 NICU patients (pre–COVID-19 patients, n=386; intra–COVID-19 patients, n=326), excluding those with COVID-19, to assess clinician networks to describe pre–COVID-19 and intra–COVID-19 teamwork structures. These 2 groups were compared using patients’ clinical characteristics, including their age, sex, race, length of stay (LOS), and discharge dispositions.

To protect patient confidentiality, analysis of EHR data was conducted at a data analysis server located at the VUMC. The EHR data used in this study were physically housed in a secure room at the VUMC’s data center. All connections made to the servers were made in an encrypted manner and used Secure Shell technology from known computers. A unique login and password were set for each authorized individual. All protected health data remained on the server, and no copy of the data was provided to unauthorized parties. The Vanderbilt institutional review board reviewed and approved the study (protocol No. 200792).

Clinicians’ EHR actions stemmed from different tasks, including conditions (eg, assessing a patient’s condition), procedures (eg, intubation), medications (eg, prescription drugs), notes (eg, progress note writing), orders (eg, laboratory test ordering), and measurements (eg, measuring blood pressure). We leveraged the actions committed to the EHRs of patients by clinicians to measure clinician-clinician connections. Prior research shows that a 1-day window can capture meaningful collaborative relationships among clinicians [12-15]. Based on their findings, we characterized a collaboration relationship (tie) between 2 clinicians as they performed actions to EHRs of the same patient within the same day (24 hours). This definition can capture different types of interactions between clinicians. The first type is the asynchronous interactions between clinicians; for instance, a clinician created a medication order at 9 AM, and another clinician reviewed and processed the order at 11 AM on the same day. Thus, the 2 clinicians had an asynchronous collaboration in terms of order creation and processing. The second type is the interactions between clinicians during shifts or handoffs, which are among the most critical aspects of collaboration owing to the medical errors that occur during the transition between clinicians [17-19,21]. For instance, 2 nurses (oncoming and ongoing) were responsible for a patient’s handoff or shift at 7 PM. The 2 nurses may perform some actions to EHRs of the patient before, during, or after the handoff/shift, which can be captured by our definition of collaboration relationship, to build a connection between them. The third type is the interactions between clinicians built on their documentation in EHRs or messages in the basket, a communication hub where clinicians can send and receive secure messages. For instance, 2 clinicians work together during patient care without interacting with EHRs, but both made some documentation later. Based on our collaboration definition, the 2 clinicians still have a connection built between them. We quantified the weight of a relation between 2 clinicians as the number of patients they co-managed on the same day, which can be learned from EHRs. We referred to each patient on that day as a patient day. The relation’s final weight is the cumulative number of patient days the 2 clinicians interacted by co-managing patients during our investigated time window (4 months). Thus, we built the pre– and intra–COVID-19 networks.

Formally, the nodes in the pre– and intra–COVID-19 networks were defined as *Z_pre_ =* {*z_1_, z_2_, …, z_p_*} and *Z_intra_ =* {*z_1_, z_2_, …, z_q_*}, respectively. To better interpret the networks, we used the clinician’s specialty (eg, respiratory therapist or NICU registered nurse) to label each node. Specialties in the pre– and intra–COVID-19 networks are referenced as *EXP_pre_ =* {*exp_1_, exp_2_, …, exp_a_*} and *EXP_intra_ =* {*exp_1_, exp_2_, …, exp_b_*}, respectively. *Z* and *EXP* are used to describe the compositions of the pre– and intra–COVID-19 networks.

**Figure 1:**
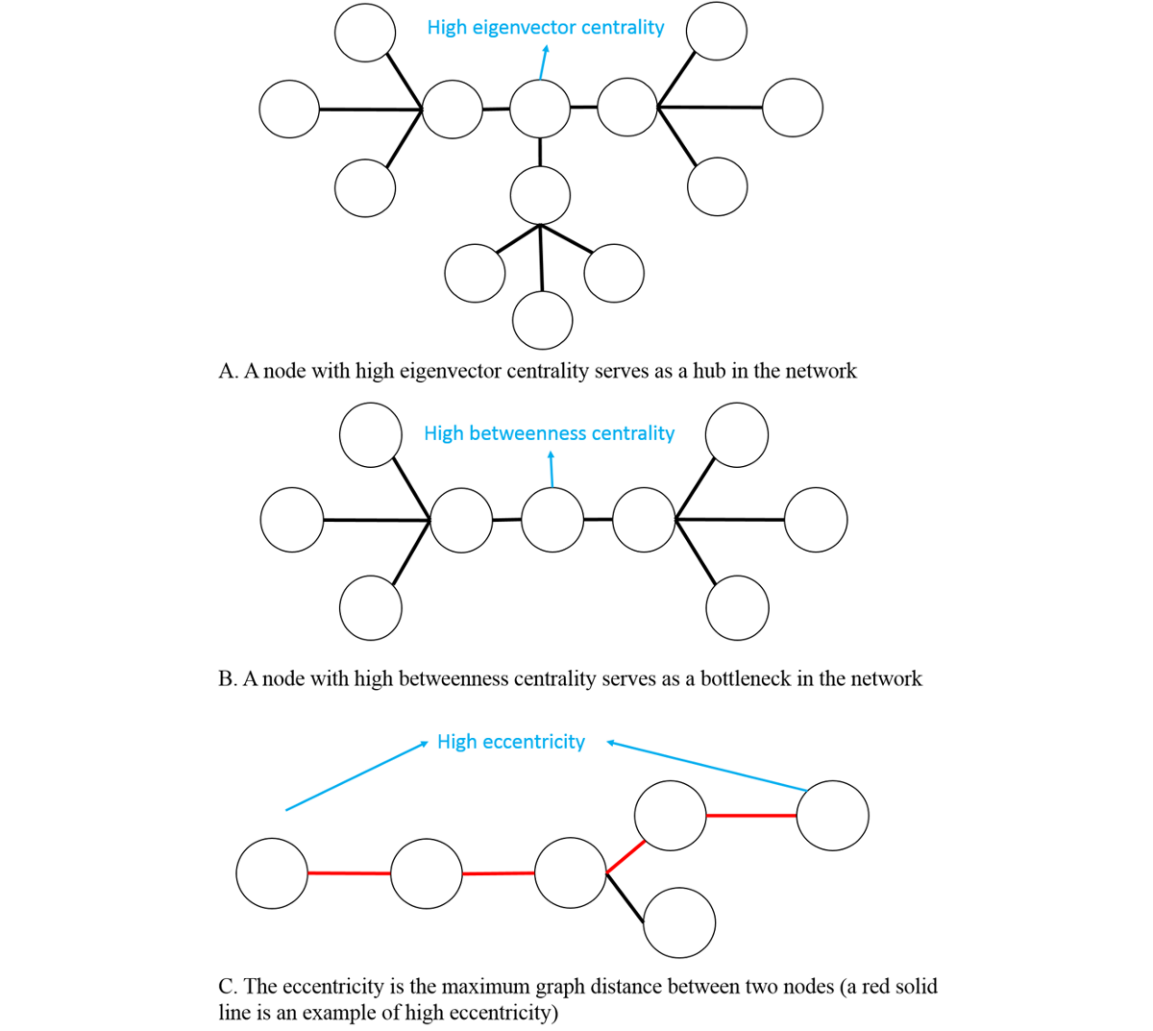
Example networks to illustrate (A) eigenvector centrality, (B) betweenness centrality, and (C) eccentricity.

We leveraged sociometric measurements, including eigenvector centrality [22], betweenness centrality [23], and eccentricity [24], to quantify the network structures. Eigenvector centrality is the measure of the influence of a node in a network [22]. In a health care EHR setting, high eigenvector centrality implies that the clinician has a very active role and serves as a hub in information sharing and dissemination. Betweenness is a measure of centrality based on the shortest paths in a network; it is calculated as the number of times a node acts as a bridge along the shortest path between two other nodes [23]. A node with higher betweenness centrality has more control in the network because owing to its shorter paths to other nodes, more information will pass through that node. Eccentricity of a node in a network is the maximum distance from the node to any other node [24]. In a clinician setting, eccentricity is the radius from one clinician to another, which is the largest distance. A larger eccentricity implies that there many more steps to share information with another clinician. Therefore, we characterized eigenvector centrality as indicating a clinician’s leadership (hub) in terms of collaboration, betweenness centrality as demonstrating a clinician cares for a wide spectrum (bridge) of patients, and eccentricity as showing the difficulty for a clinician to collaborate with others. Figure 1 shows 3 networks to illustrate each of the 3 sociometric measurements, respectively. We used Gephi, an open-source network analysis and visualization software package [25], to calculate eigenvector centrality, betweenness centrality, and eccentricity for each of the nodes in the pre– and intra–COVID-19 networks.

We investigated whether the differences in the clinician leadership, who care for a wide spectrum of patients, and collaboration difficulty are statistically different between pre– and intra–COVID-19 networks. In addition, we investigated changes in 2 outcome metrics, including LOS and discharge dispositions, from the pre– to the intra–COVID-19 periods. We applied Mann-Whitney *U* tests at a 95% CI to account for the non-Gaussian distribution of the sociometric measurements and outcomes. Since the pre– and intra–COVID-19 networks are composed of clinicians with different specialties, we compared the differences at both network (entire network) and specialty (each specialty) levels. We applied a Bonferroni correction to account for multiple hypothesis testing (eg, pairwise test for the specialty-level comparisons).

For each investigated specialty (eg, NICU nurse), we investigated whether clinicians affiliated with the specialty in the pre–COVID-19 network have significantly higher values of eigenvector centrality, betweenness centrality, or eccentricity than those affiliated with the same specialty in the intra–COVID-19 network. We focused on the 6 specialties that play essential roles in NICU care, which included NICU nurses, nurse practitioners, residents, respiratory therapists, cardiac ICU nurses, and neonatologists. For an investigated specialty, we created 2 arrays, one with values of a sociometric measurement (eg, eigenvector centrality) of nodes (clinicians) affiliated with the specialty in the pre–COVID-19 network and the other for the values of the same sociometric measurement of clinicians affiliated with the same specialty in the intra–COVID-19 network.

We further tested the differences in the eigenvector centrality, betweenness centrality, and eccentricity between pre– and intra–COVID-19 networks using all specialties in the 2 networks. The hypothesis test is of the following form: there are significant differences in eigenvector centrality, betweenness centrality, or eccentricity between the pre– and intra–COVID-19 networks. Within a network, we measured eigenvector centrality, betweenness centrality, or eccentricity for a specialty by calculating the mean values of eigenvector centrality, betweenness centrality, or eccentricity of all clinicians affiliated with that specialty in the network. For pre– or intra–COVID-19 networks, we developed an array of specialties, whose cell value is the value of a specialty’s sociometric.

The data sets generated and analyzed during the current study are not publicly available in order to maintain the privacy of the patient information investigated in this study, but they are available from the corresponding author on reasonable request.

## Results

The pre– and intra–COVID-19 patient groups share similar distributions (Table 1) in sex (~0 difference), race (4% difference in White, and 3% difference in African American), LOS (IQR difference in 1.5 days), and discharge dispositions (~0 difference in home, 2% difference in expired, and 2% difference in others). There were no significant differences in patient demographics and outcomes between the 2 groups.

There are several notable findings in network analysis to highlight. First, the intra–COVID-19 NICU structure had a higher eccentricity (collaboration difficulty) than the pre–COVID-19 (median 3 vs 4; *P*<.001). Second, NICU nurses had a lower eigenvector centrality (leadership in collaboration) in the intra–COVID-19 structure than the pre–COVID-19 (median 0.183 vs 0.087; *P*<.001). Third, neonatology physicians had a higher betweenness centrality (care for a wider spectrum of patients) in the intra–COVID-19 than in the pre–COVID-19 structure (median 0.0001 vs 0.005; *P*<.005).

**Table 1:** Characteristics of patients admitted to the neonatal intensive care unit in the pre– and intra–COVID-19 groups (N=712).

Characteristics			Pre–COVID-19 group (n=386)	Intra–COVID-19 group (n=326)
**Demographic information**				
	Age (days), median (IQR)		0.0 (0.0-0.0); maximum, 149.0	0.0 (0.0-0.0); max-imum, 97.0
	**Sex, n (%)**			
		Female	159 (41.2)	134 (41.1)
		Male	159 (41.2)	134 (41.1)
	**Race, n (%)**			
		White	258 (66.8)	231 (70.9)
		African American	64 (16.6)	43 (13.2)
		Asian	16 (4.1)	6 (1.8)
		Other	47 (12.2)	46 (14.1)
	**Ethnicity, n (%)**			
		Non-Hispanic	331 (85.8)	283 (86.8)
		Latino	50 (13.0)	32 (9.8)
		Unknown	5 (1.3)	11 (3.4)
**Outcomes**				
	Length of stay, median (IQR)		10.0 (4.0-20.0)	9.0 (3.3-21.8)
	**Hospital disposition, n (%)**			
		Home	354 (91.7)	299 (91.7)
		Expired	27 (7.0)	16 (4.9)
		Hospice	2 (0.5)	2 (0.6)
		Short-term hospital	3 (0.8)	8 (2.4)
		Others	0 (0)	1 (0.3)

**Figure 2:**
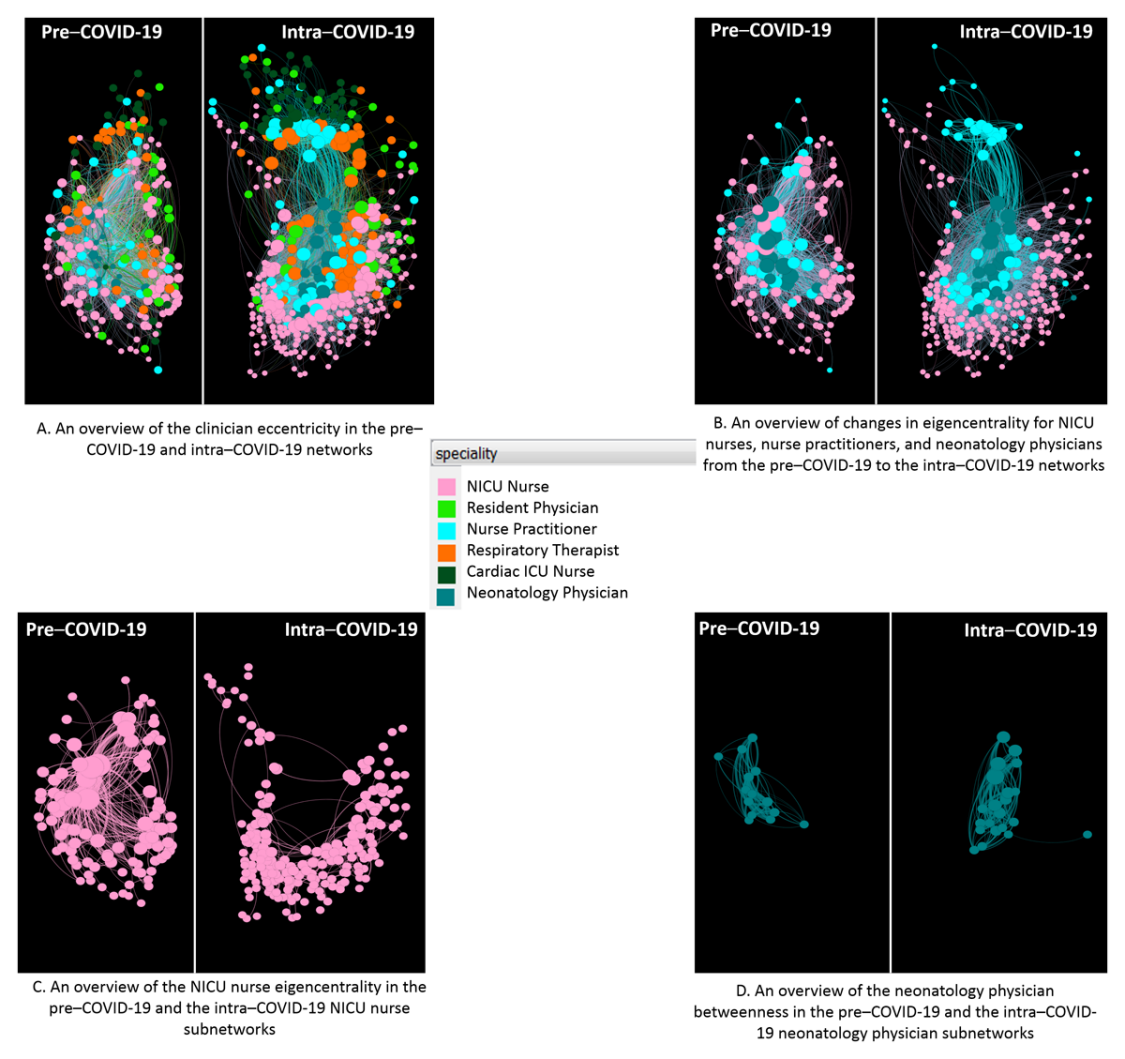
Overviews of the eccentricity of the 6 types of clinicians in (A) the pre– and intra–COVID-19 networks, (B) overviews of the eigenvector centrality of nurses, nurse practitioners, and neonatology physicians in the pre– and intra–COVID-19 networks, (C) subnetworks of NICU nurses and their eigenvector centrality in the pre– and intra–COVID-19 settings, and (D) subnetworks of neonatology physicians and their betweenness centrality in the pre– and intra–COVID-19 settings. The legend in the center shows the colors of the 6 roles, which have the largest number of clinicians affiliated. The eccentricity is directly correlated with the corresponding node size in (A), the eigenvector centrality is directly correlated with the corresponding node size in (B) and (C), and the betweenness centrality is directly correlated with the corresponding node size in (D). ICU: intensive care unit, NICU: neonatal intensive care unit.

Figure 2 shows the pre– and intra–COVID-19 networks from different perspectives. Figure 2A shows that the intra–COVID-19 network is larger, implying that the 6 types of clinicians are likely to be more difficult to collaborate with others (higher eccentricity) in the intra–COVID-19 NICU care than those in the pre–COVID-19 NICU care (median 4 vs 3; *P*<.001). This higher eccentricity in intra–COVID-19 implies that there is more distance from one node to any other node within the network. Compared to nurse practitioners and neonatology physicians, nurses’ leadership (eigenvector centrality) reduced from the pre– to intra–COVID-19 networks (median 0.183 vs 0.087; *P*<.001), as shown in Figure 2B. This reduced leadership implies that nurses were less active during the intra–COVID-19 period. Nurses do not occupy central positions in the intra–COVID-19 network, and they have more connections in the pre–COVID-19 period than in the intra–COVID-19 period, as shown in Figure 2C. Neonatology physicians care for a wider spectrum of patients (high betweenness centrality) in intra–COVID-19 NICU care than those in pre–COVID-19 NICU care (median 0.005 vs 0.0001; *P*<.001), as shown in Figure 2D. Therefore, during the COVID-19 pandemic, neonatology physicians have a higher number of shorter paths to other nodes within the network.

## Discussion

### Principal Findings

To follow the COVID-19 physical distancing policy, the VUMC involves new EHR use practices to provide care for patients during the COVID-19 pandemic. The collaboration difficulty (increased eccentricity) can be a potential problem in the new EHR use practices. In the post–COVID-19 era, when HCOs plan to promote more collaboration in virtual platforms, they may need to develop staffing strategies to reduce the collaboration difficulty in EHRs.

Neonatologists care for a wider spectrum of patients (higher betweenness centrality) when using EHRs during the COVID-19 pandemic. HCOs may need to develop educational strategies to promote EHR collaboration between neonatologists and other clinicians to improve teamwork efficiency and NICU outcomes in the post–COVID-19 era. NICU nurses have reduced leadership (lower eigenvector centrality) in cooperation, suggesting that increased EHR use may reduce nurses’ workload in the collaboration.

Our results for network analysis of collaboration structures demonstrate changes in virtual care from the pre– to the intra–COVID-19 periods. Findings in prior literature also reflect our results. Reeves et al [26] reported the increasing utilization of electronic check-in, standard ordering and documentation, secure messaging, real-time data analytics, and telemedicine during the COVID-19 pandemic, compared to those before the pandemic. Furthermore, Wosik et al [5] examined how people, processes, and technology (EHRs) work together to support a successful virtual care transformation.

Our results show there are no significant changes in LOS and discharge dispositions, which indicates the changes in clinicians’ connections to protect patients and health care professionals during the COVID-19 pandemic have few impacts on the 2 outcomes. However, the effect of the changes on the satisfaction of patients’ families and clinicians has not been investigated. Although our results are dependent on an investigation of 1 health system, the network analysis methods used in our study could be used to extrapolate results in different countries with different health care systems.

### Limitations

There are several limitations in this pilot study, which should be recognized. The characteristics of the NICU structures learned from this single-center analysis could provide some reference for other HCOs when they assess their NICU structures. However, VUMC NICU is a highly collaborative environment, which should be considered when interpreting the results and findings. Second, there is a lack of standard terminology for characterizing NICU specialties. Common data models for clinician types would improve the quality of our study and assist in the transition of our methodology to other institutions. Third, we assumed that 2 clinicians have a connection when they commit actions to the EHRs of patients on the same day. Although such an assumption can capture collaboration relationships among clinicians, it may also reveal many spurious relationships. This assumption also only looks at clinician connections within the EHR; failing to identify in-person (eg, discussing patient results with another clinician) or virtual interactions (eg, Zoom meetings). Furthermore, the connection between the 2 clinicians indicates potential collaboration (information sharing) rather than actual collaboration. Finally, fine-grained EHR actions are required to add contextual information to the relations between clinicians. For instance, the connection between a nurse and a consultant is created on the basis of their communications in flowsheets data.

### Conclusions

The developed network methods can be effective tools to assess differences in collaboration structures in current and future disruptions in health care delivery (eg, pandemics) and major transitions (physical to virtual collaboration) adopted by HCOs. The methods and the results of our study can also be used to analyze clinician’s leadership, collaboration difficulty, and broad skill sets in different health care studies. In future studies, recruiting subject matter experts (eg, clinics) to evaluate the learned connections and ICU structures will be required to validate the results. This knowledge of connections among clinicians can assist HCOs with developing more specific staffing strategies, which may improve care quality and patient outcomes.

### Clinical Trial Registration

Non–clinical trial study.

## Appendix

Multimedia Appendix 1 Reporting checklist for quality improvement in health care.

## Abbreviations

EHR: electronic health record
HCO: health care organization
ICU: intensive care unit
LOS: length of stay
NICU: neonatal intensive care unit
VUMC: Vanderbilt University Medical Center
